# A Radiomics Nomogram for Classifying Hematoma Entities in Acute Spontaneous Intracerebral Hemorrhage on Non-contrast-Enhanced Computed Tomography

**DOI:** 10.3389/fnins.2022.837041

**Published:** 2022-06-10

**Authors:** Jia Wang, Xing Xiong, Jing Ye, Yang Yang, Jie He, Juan Liu, Yi-Li Yin

**Affiliations:** ^1^Department of Radiology, Northern Jiangsu People’s Hospital, Yangzhou, China; ^2^Department of Radiology, The First Affiliated Hospital of Soochow University, Suzhou, China

**Keywords:** radiomics, nomogram, non-contrast-enhanced computed tomography, intracerebral hemorrhage, vascular malformations-related hemorrhage

## Abstract

**Aim:**

To develop and validate a radiomics nomogram on non-contrast-enhanced computed tomography (NECT) for classifying hematoma entities in patients with acute spontaneous intracerebral hemorrhage (ICH).

**Materials and Methods:**

One hundred and thirty-five patients with acute intraparenchymal hematomas and baseline NECT scans were retrospectively analyzed, i.e., 52 patients with vascular malformation-related hemorrhage (VMH) and 83 patients with primary intracerebral hemorrhage (PICH). The patients were divided into training and validation cohorts in a 7:3 ratio with a random seed. After extracting the radiomics features of hematomas from baseline NECT, the least absolute shrinkage and selection operator (LASSO) regression was applied to select features and construct the radiomics signature. Multivariate logistic regression analysis was used to determine the independent clinical-radiological risk factors, and a clinical model was constructed. A predictive radiomics nomogram was generated by incorporating radiomics signature and clinical-radiological risk factors. Nomogram performance was assessed in the training cohort and tested in the validation cohort. The capability of models was compared by calibration, discrimination, and clinical benefit.

**Results:**

Six features were selected to establish radiomics signature *via* LASSO regression. The clinical model was constructed with the combination of age [odds ratio (OR): 6.731; 95% confidence interval (CI): 2.209–20.508] and hemorrhage location (OR: 0.089; 95% CI: 0.028–0.281). Radiomics nomogram [area under the curve (AUC), 0.912 and 0.919] that incorporated age, location, and radiomics signature outperformed the clinical model (AUC, 0.816 and 0.779) and signature (AUC, 0.857 and 0.810) in the training cohort and validation cohorts, respectively. Good calibration and clinical benefit of nomogram were achieved in the training and validation cohorts.

**Conclusion:**

Non-contrast-enhanced computed tomography-based radiomics nomogram can predict the individualized risk of VMH in patients with acute ICH.

## Introduction

Spontaneous intracerebral hemorrhage (ICH) is defined as intraparenchymal hemorrhage caused by vascular rupture, which can be divided into primary hemorrhage and secondary hemorrhage (Meretoja et al., 2012). Primary intracerebral hemorrhage (PICH) is mainly due to spontaneous rupture of blood vessels caused by hypertension, arteriosclerosis, or amyloidosis, while secondary cerebral hemorrhage generally refers to the existence of secondary cause, such as the hemorrhagic transformation of ischemic stroke, cerebral venous thrombosis, tumors, and vascular malformation-related hemorrhage (VMH). For patients suspected of acute ICH, non-contrast-enhanced computed tomography (NECT) scan remains the first choice of imaging during a clinical emergency, which can effectively reflect the location, volume, and surrounding brain tissue structure of hemorrhage (Thabet et al., 2017). Several studies have shown a major difference in treatment and prognosis between VMH and PICH (Barone et al., 2017; Fukuda et al., 2017). For PICH, the primary strategy is to monitor vital signs and hemorrhage changes, stabilize blood pressure, and prevent further bleeding. Unless the patient is suffering from severe intracranial hypertension or cerebral hernia, surgical treatment should not be considered. Ruptured VMH can easily re-hemorrhage, so surgical resection or interventional embolization has a positive effect on the prognosis of patients.

It is crucial to distinguish VMH from PICH in the early stage. However, it is difficult and equivocal to distinguish hemorrhage types by the naked eye on NECT images. As a quantitative imaging research method, radiomics can non-invasively evaluate regional tissue heterogeneity at the millimeter scale, which has been confirmed in tumor imaging (Lambin et al., 2017). However, the radiomics research in hemorrhagic diseases is limited, and no study has reported a NECT-based radiomics nomogram to differentiate VMH from PICH.

In this study, we hypothesized that VMH contains unique NECT-based radiomics features, and we aimed to develop and validate a radiomics nomogram for the classification of VMH, to facilitate individualized treatment strategies for patients with acute ICH.

## Materials and Methods

### Subjects

This study was approved by the Ethics Committee of the local institution, and informed consent was waived off. The patients were retrospectively analyzed between October 2017 and September 2020, i.e., 333 patients with acute ICH who received baseline NECT scan after symptom onset. The inclusion criteria were as follows: (1) acute, non-traumatic intraparenchymal hemorrhage; (2) NECT scan within 6 h of the onset of ICH symptoms; and (3) complete medical records. Patients with the following conditions were excluded: (1) acute brain injury (*n* = 56); (2) isolated intraventricular hemorrhage, subarachnoid hemorrhage, epidural hemorrhage, or venous sinus embolism (*n* = 43); (3) hemorrhage related to tumor, aneurysm, or ischemic stroke (*n* = 36); (4) multiple hemorrhages (*n* = 9); (5) baseline NECT scan after 6 h of the onset (*n* = 39); and (6) serious image artifacts (*n* = 15).

A total of 135 consecutive patients (95 men and 40 women; mean age, 54.22 ± 15.38 years) were enrolled, and their demographic information was collected from medical records. VMH was diagnosed according to post-digital subtraction angiography (DSA) and/or surgery and repeated DSAs were operated if considered necessary. Among them, 41 were confirmed as arteriovenous malformations, 7 as cavernous malformations, and 4 as arteriovenous fistula. The database was divided into a training cohort (*n* = 95; 65 men and 30 women; mean age, 54.06 ± 15.70 years) and a validation cohort (*n* = 40; 30 men and 10 women; mean age, 54.60 ± 14.61 years) in a 7:3 ratio with a random seed. Models were developed in the training cohort and independently tested in the validation cohort.

### CT Imaging

All patients underwent baseline head NECT scan using a GE Gemstone Scanner. The scan protocol was as follows: (1) tube voltage, 120 kV; (2) tube current, 360 mA; (3) field of view, 320 mm; (4) matrix, 512 × 512; and (5) slice thickness, 5 mm.

### Qualitative Analysis of Non-contrast-Enhanced Computed Tomography Images

All CT images were analyzed by two radiologists with one having experience of 7 years and another with 5 years independently. They were blinded to the clinical information. The two radiologists evaluated the following image features of hemorrhage: (1) location (deep/lobar) and (2) shape (regular or irregular). A discussion was carried out to achieve consensus in case of discrepancy.

### Clinical-Radiological Risk Factors

The univariate analysis was used to assess the single clinical and NECT features for discriminating VMH from PICH in the training cohort, and a multivariate logistic regression analysis with significant variables was performed to determine the potential risk factors of VMH.

### Image Processing and Segmentation

The voxel-based segmentation of hemorrhage was performed using 3D-Slicer software (version 3.6.0^1^) by one radiologist. Regions of interest (ROI) were semi-automatically delineated on each slice of the CT image containing the entire lesion. The segmentations were then validated by another radiologist in a cohort of 30 randomly selected patients. All images were resampled to 1 mm × 1 mm × 1 mm voxel and normalized by histogram matching to eliminate the intensity difference.

### Extraction of Radiomics Feature

Prior to extracting the features, image filtration was implemented on the original image with wavelet (wavelet-LHL, wavelet-LHH, wavelet-HLL, wavelet-LLH, wavelet-HLH, wavelet-HHH, wavelet-HHL, and wavelet-LLL) and Laplacian of Gaussian (LoG) transform. The log filtration was calculated with operator values of 1 mm (fine), 3 mm (medium), and 5 mm (coarse), respectively. A set of 1,130 radiomics features was extracted from each original and filtered segmentation and divided into five groups: (1) intensity (histogram-derived first-order statistics (*n* = 18); (2) shape (*n* = 14); (3) textural matrix [i.e., the gray-level co-occurrence matrix (GLCM), gray-level run-length matrix (GLRLM), gray-level size-zone matrix (GLSZM), and the neighborhood gray-tone difference matrix (NGTDM), *n* = 75]; (4) wavelet-based transform (*n* = 744), and (5) log-based transform (*n* = 279). All features were automatically extracted from the volumes of interest (VOIs), which contain 3D information on hematoma using PyRadiomics, and the detailed descriptions of radiomics features could be found on the https://pyradiomics.readthedocs.io/en/latest/index.html.

### Selection of Radiomics Feature

**FIGURE 1:**
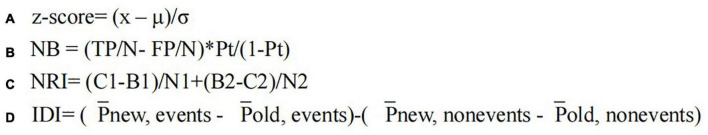
Formulas of *z*-score **(A)**, net benefit **(B)**, net reclassification index **(C)**, and integrated discrimination index **(D)**. *x*, the feature value; μ, the average of the feature values among all VOIs; σ, the corresponding standard deviation; NB, net benefit; TP, true positive; FP, false positive; *N*, total cases; Pt, probability threshold; NRI, net reclassification index; C1, B1, the cases predicted by the new and old model in the event occurrence group; B2, C2, the cases predicted by the new and old model in the event incurrence group; N1, N2, the cases in the event occurrence and incurrence group; IDI, integrated discrimination index; −Pnew, events,−Pold, events, the mean of the probability of disease predicted by the new and old model for each individual in the event occurrence group; Pnew, non-events,−Pold, non-events, the mean of the probability of disease predicted by the new and old model for each individual in the event incurrence group.

Inter-class correlation (ICC) coefficient was calculated for each radiomics feature, and a single-factor logistic regression analysis was carried out to select highly significant and correlated features. Only the features with ICC > 0.8 and *p* < 0.1 were considered as high repeatability and correlated, which were included for subsequent analysis. Then, the feature values of all VOIs were normalized with *z*-score normalization (Figure 1A) to eliminate the unit limit for each feature. The least absolute contraction and selection operator (LASSO) was applied to select features to reduce the redundancy and overfitting in the training cohort. The set of non-zero coefficient features was chosen with optimal λ value, which was determined by the area under the curve (AUC) of fivefold cross-validation. The radiomics score (Rad-score) of each patient was calculated using the linear fitting of selected features, and the radiomics signature was constructed. The formula was as follows: $\mathrm{Rad}-\mathrm{score}=\alpha +\sum_{1}^{i}\beta_{i}\,X_{i}$ (α, the intercept; β_*i*_, the value of radiomics feature selected by LASSO; β_*i*_, the corresponding coefficients).

### Construction and Evaluation of the Prediction Model

After the selection of potential clinical-radiological risk factors of VMH based on the multivariate logistic regression analysis, the clinical model in the training cohort was built. Then, the radiomics nomogram model incorporating the clinical-radiological risk factors and radiomics signature were also constructed. The calibration curves and the Hosmer–Lemeshow test were performed to evaluate the goodness-of-fit of the nomogram. Receiver operating characteristic (ROC) curve analysis was used to evaluate the discrimination performance of the models. Decision curve analysis (DCA) was performed to determine the clinical benefit of the models by calculating their net benefits (Figure 1B). The categorical net reclassification index (NRI, three risk categories: <0.5, 0.5–0.8, and >0.8, Figure 1C) and total integrated discrimination index (IDI, Figure 1D) were calculated to evaluate the prediction improvement of the radiomics nomogram when compared to clinical-radiological factors.

### Statistical Analysis

Student’s *t*-test, the Mann–Whitney *U* test, the Chi-square test, and Fisher’s exact test were used for univariate analysis, as appropriate. The ROC curves were compared using the DeLong test. The statistical analysis was performed using R (version 3.6.2, Boston, MA, United States) and SPSS software (version 22, Chicago, IL, United States). Two-tailed values of *p* < 0.05 were considered as statistically significant.

## Results

### Patient Characteristics

There were significant differences in age, history of hypertension, and location between the VMH group and the PICH group in the training cohorts (*p* < 0.001), but there were no significant differences in gender, history of smoking, diabetes, alcohol consumption, and coagulopathy or shape (*p* > 0.05; Table 1). No differences were found in clinical-radiological characteristics between the training and validation cohorts (*p* = 0.137–0.922).

**TABLE 1:** Demographic data and NECT features.

Variables	Training cohort (*n* = 95)			Validation cohort (*n* = 40)		
	VMH	PICH	*p*	VMH	PICH	*p*
Gender (male/female)	26/10	39/20	0.534	11/5	19/5	0.456
Age (years, mean ± SD)	42.36 ± 17.03	61.20 ± 14.89	<0.001	41.75 ± 15.70	63.17 ± 13.88	<0.001
Hypertension (P/N)	13/23	42/17	<0.001	2/14	16/8	0.001
Diabetes (P/N)	3/33	10/49	0.358	1/15	3/21	0.638
Smoking (P/N)	2/34	11/48	0.122	3/13	6/18	0.717
Alcohol consumption (P/N)	1/35	7/52	0.252	1/15	4/20	0.631
Coagulopathy (P/N)	0/36	3/56	0.286	0/16	3/21	0.262
Location (deep/lobar)	7/29	39/20	<0.001	5/11	14/10	0.093
Shape (regular/irregular)	20/16	36/23	0.600	5/11	13/11	0.154

*NECT, non-contrast-enhanced CT; VMH, vascular malformations-related hemorrhage; PICH, primary intracerebral hemorrhage; P, positive; N, negative.*

### Clinical Model Construction

Multivariable analysis showed that age (subdivided into ≥50 and <50 years old classes, *p* = 0.001) and location (*p* < 0.001) were independent risk factors for clinical model construction. Patients with younger age [odds ratio (OR): 6.731; 95% confidence interval (CI): 2.209–20.508] or hemorrhages located in the lobar (OR: 0.089; 95% CI: 0.028–0.281) were likely to be VMH.

### Radiomics Signature Construction

**FIGURE 2:**
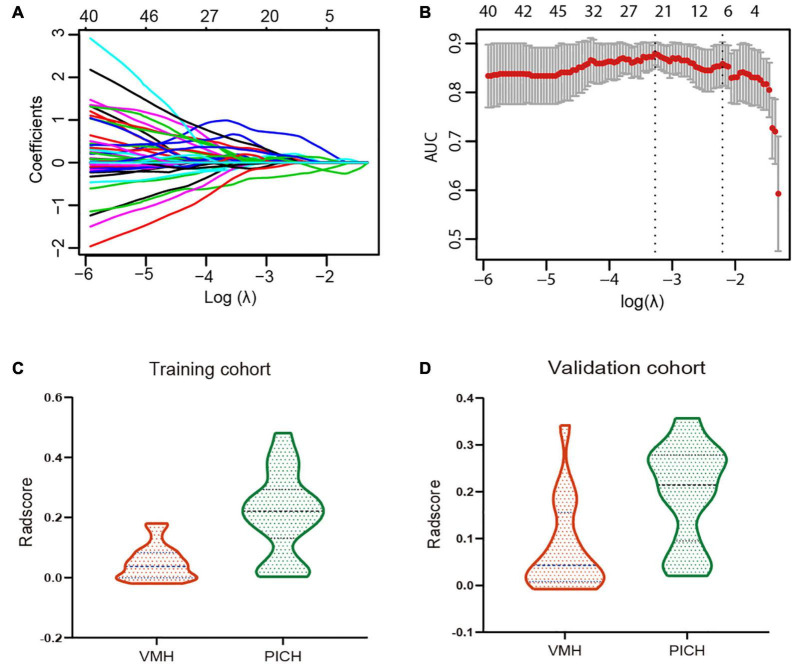
**(A)** Tuning parameter (λ) selection in the least absolute shrinkage and selection operator (LASSO) regression used fivefold cross-validation *via* one standard error (SE) of the minimum criteria. The optimal λ value was 0.1212 with log(λ) = 2.1095. **(B)** LASSO regression coefficient of the 532 radiomics features generated vs. the selected log(λ) value. **(C,D)** The violin plots for radiomics signature in the training and validation cohorts, categorized by the vascular malformations-related hemorrhage (VMH) and PICH groups.

Of the 1,130 radiomics features extracted from NECT images, 532 features were demonstrated with ICC > 0.8 and *p* < 0.1. Six VMH-related features with non-zero coefficients were selected using a LASSO logistic regression for Rad-score calculation, and a radiomics signature was constructed (Figures 2A,B). The selected radiomics features and the corresponding coefficients are displayed in Table 2. The Rad-score differed significantly between the VMH and PICH groups in both training (*p* < 0.001) and validation (*p* < 0.05) cohorts (Figures 2C,D). Patients with VMH generally had lower Rad-scores than those with PICH.

**TABLE 2:** The selected radiomics features and coefficients.

Image	Class	Name	Coefficients
Sigma-5 mm	GLCM	Lmc2	−0.1157
Sigma-5 mm	GLCM	Lmc1	0.4231
Wavelet-HLL	GLCM	Idn	−0.2140
Wavelet-LHL	GLCM	Correlation	0.0068
Wavelet-LLL	GLSZM	GrayLevelNonUniformityNormalized	0.1238
Original	GLSZM	LowGrayLevelZoneEmphasis	0.0331

*GLCM, gray level co-occurrence matrix; GLSZM, gray level size zone matrix.*

### The Radiomics Nomogram Development and Model Assessment

**FIGURE 3:**
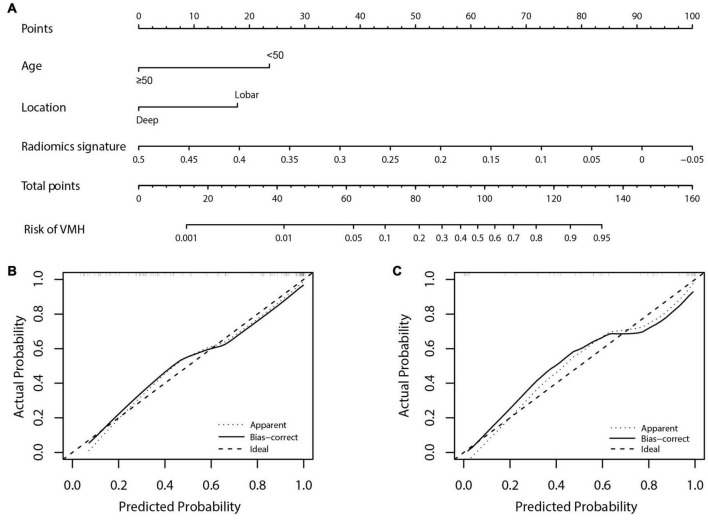
The radiomics nomogram for predicting the status of hemorrhage and calibration curves. **(A)** The radiomics nomogram, combining age, location, and radiomics signature were developed in the training cohort. Patients with younger age (<50 years) or hemorrhages located in lobes and the negative radiomics signature were more likely to be vascular malformations-related hemorrhage (VMH). **(B,C)** Calibration curves for the radiomics nomogram in the training and validation cohorts. Calibration curves indicated the agreement of the nomogram between the predicted risk of VMH and actual status. The 45° gray dotted line represents the ideal prediction and the black curve is the predictive performance of the nomogram. The closer the black curve is to the gray dotted line, the better is the predictive efficacy of the nomogram.

Age, location, and radiomics signature were incorporated into the development of radiomics nomogram (Figure 3A). Patients with younger age (<50 years) or hemorrhages located in lobes and the negative radiomics signature were more likely to be VMH. Figure 3B shows that the calibration curve of the nomogram fitted well with the actual status. The Hosmer–Lemeshow test showed good calibration in the training cohort (*p* = 0.914).

**FIGURE 4:**
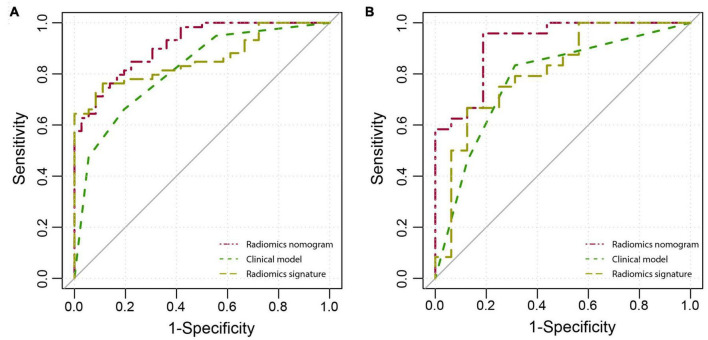
The receiver operator characteristic (ROC) curves of the radiomics nomogram, clinical model, and radiomics signature in the training **(A)** and validation **(B)** cohorts.

The diagnostic performance of each model is summarized in Table 3, and the ROC curves are shown in Figure 4. Compared with radiomics signature (AUC: 0.857; 95% CI: 0.771–0.921; sensitivity, 0.763; and specificity, 0.889) and clinical model (AUC: 0.816; 95% CI: 0.723–0.888; sensitivity, 0.661; and specificity, 0.806), the radiomics nomogram (AUC: 0.912; 95% CI: 0.836–0.960; sensitivity, 0.746; and specificity, 0.889) showed better performance in differentiating VMH from PICH. The DeLong test showed that the performance of the radiomics nomogram had a statistically significant improvement (*p* < 0.05).

**TABLE 3:** Predictive performance of radiomics nomogram, radiomics signature, and clinical model.

Model	Training cohort				Validation cohort			
	AUC (95% CI)	SEN	SPE	Youden index	AUC (95% CI)	SEN	SPE	Youden index
Clinical model	0.816 (0.723–0.888)	0.661	0.806	0.467	0.779 (0.620–0.894)	0.833	0.688	0.521
Radiomics signature	0.857 (0.771–0.921)	0.763	0.889	0.652	0.810 (0.655–0.916)	0.667	0.875	0.542
Radiomics nomogram	0.912 (0.836–0.960)	0.746	0.889	0.635	0.919 (0.788–0.982)	0.958	0.813	0.771

*AUC, area under the curve; CI, confidence interval; SEN, sensitivity; SPE, specificity.*

### Model Validation

Figure 3C shows the calibration curve of the nomogram in the validation cohort with a non-significant Hosmer–Lemeshow test statistic (*p* = 0.720). The AUC of the radiomics nomogram (AUC: 0.919; 95% CI: 0.788–0.982) was higher than that of the clinical model (AUC: 0.779; 95% CI: 0.655–0.916) and radiomics signature (AUC: 0.810; 95% CI: 0.788–0.982) in the validation cohort.

**FIGURE 5:**
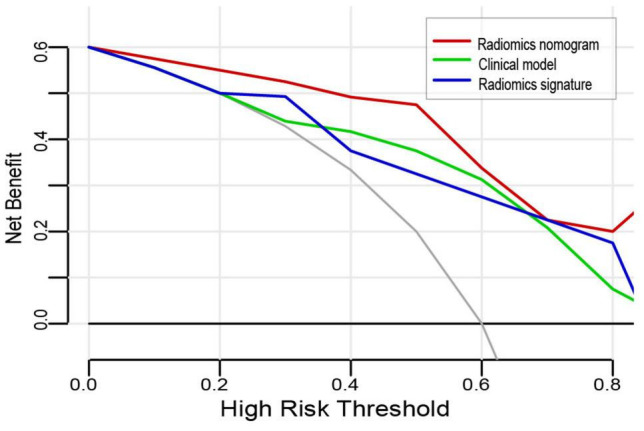
Decision curve analysis for the three models. The *y*-axis indicates the net benefit; the *x*-axis indicates the threshold probability. The red line, green line, and blue line represent the net benefit of the radiomics nomogram, clinical model, and radiomics signature, respectively. The radiomics nomogram had the highest net benefit compared with the other two models when the threshold probability was <0.8 at which a patient would be diagnosed as vascular malformations-related hemorrhage (VMH).

The DCA for the three models is shown in Figure 5. The curve showed that the radiomics nomogram had a higher overall net benefit in differentiating VMH from PICH than the clinical model and radiomics signature, within the threshold probability <0.8.

The discrimination measures confirmed that adding radiomics signature to clinical-radiological factors significantly improved reclassification for predicting hemorrhage status, with NRI and IDI of 0.435 (95% CI: 0.178–0.692, *p* < 0.001) and 0.170 (95% CI: 0.092–0.247, *p* < 0.001), 0.313 (95% CI: −0.049 to 0.674, *p* < 0.001), and 0.250 (95% CI: 0.103–0.3961, *p* < 0.001) in the training and validation cohorts, respectively.

## Discussion

In this study, we constructed and evaluated three predictive models to non-invasively distinguish VMH from PICH, such as the clinical model, radiomics signature, and radiomics nomogram. The radiomics nomogram incorporated age, hemorrhage location, and radiomics signature derived from NECT and showed satisfactory performance for predicting VMH. Thus, it provides a straightforward and safe auxiliary approach for a clinical emergency.

Patients with VMH-related hemorrhage have lower inpatient mortality and are more likely to have a favorable discharge disposition as compared to patients with PICH (Murthy et al., 2017). The timely surgical or interventional practice has a positive impact on the outcomes of patients with VMH, otherwise, the effect worsens after 48 h of the onset of symptoms. Therefore, patients with VMH should be screened as soon as possible for further operation, instead of receiving conservative treatment as the patients with PICH. The PICH is diagnosed by excluding a thorough investigation for secondary structural causes of ICH. Older age, deep location, and history of hypertension are usually considered a diagnostic basis of PICH, although cerebral angiography studies suggest that these imaging and clinical features are not always reliable indicators in selecting patients for further therapy, and patients with these features may have coexisting vascular abnormalities (Josephson et al., 2014; van Asch et al., 2015).

Prior to radiomics analysis, we evaluated the clinical-radiological factors. In our study, age and hemorrhage location were independent risk factors for distinguishing PICH and VMH, similar to the findings of Murthy et al. (2017). Several studies have shown that when compared with PICH, the VMH tended to be more irregular, and the bottom of the lesion was usually located on the cortex, with the tip of the lesion pointed to the lateral ventricle (Wagle et al., 1984). Unexpectedly, in our study, the shape of the hemorrhage was failed to show enough predictive strength based on univariable association with VMH. This may be due to the evaluation of shape (regular/irregular) being influenced by the reader’s academic background and experience. Moreover, the descriptors of two-/three-dimensional size and shape of lesions were included in radiomics features. For example, sphericity is a measure of the roundness of the shape of the region relative to a sphere (Lorensen et al., 2020). Hence, the integration of shape described by radiomics features can decrease bias when compared with visual evaluation in theory, which makes the exclusion of this variable an appropriate strategy for model development.

Radiomics, using different machine learning methods to construct predictive models, can non-invasively reflect the internal heterogeneity of lesions to achieve early diagnosis, differential diagnosis, and treatment response monitoring. This method has been widely studied in various tumor-related research studies (Kocak et al., 2018; Artzi et al., 2019; Shu et al., 2019). Compared with PICH, the internal composition and morphology of VMH can be more complex and heterogeneous due to the existence of malformed blood vessels, which can be reflected by radiomics features. So far, radiomics analysis of vascular diseases has mainly focused on the identification and stratification of stability of vessel plaque (Shi et al., 2018; Kolossvary et al., 2019), the prediction of cerebral hemorrhage expansion (Ma et al., 2019; Xie et al., 2020; Xu et al., 2020), and discrimination of tumorous ICH from benign causes (Choi et al., 2015; Nawabi et al., 2020). The current studies showed that radiomics features may have the advantages of a higher resolution and objectively quantify the heterogeneity of hematoma. In our study, six radiomics features belonging to the GLSZM and GLCM that described the heterogeneity of the hematoma may be associated with VMH.

To the best of our knowledge, only one reported study applied radiomics analysis to distinguish the hemorrhage caused by arteriovenous malformation and other etiologies (Zhang et al., 2019). Zhang et al. (2019) constructed 88 predictive models by the combination of 11 feature-selection methods and 8 classifiers predictive models, and then the optimal one was selected and evaluated. However, they included fewer radiomics features. Moreover, CT imaging features and clinical characteristics were excluded from their study. In our study, we extracted and analyzed 1,130 radiomics features, which can more comprehensively describe the internal heterogeneity of hemorrhage. After processing, five of the six optimal features were derived from LoG and wavelet filtration, which cannot be obtained by conventional texture analysis. As a result, with the aid ofmodeling NECT-based radiomics, the post-processing and reporting time are limited to 5 min. More importantly, the radiomics nomogram can generate an individual probability of VMH with its visual scoring system, which meets the requirement of personalized medicine. Additionally, DCA, NRI, and IDI analyses were applied in the current study to assess the clinical improvement of radiomics nomogram-assisted decisions on patient outcomes. The DCA showed that using the radiomics nomogram to predict VMH obtains more benefits than either the treat-all-patients or the treat-none scheme. The NRI and IDI analyses confirmed the reclassification improvement by adding radiomics signature to clinical-radiological factors.

The ability of radiomics model to distinguish different hematoma types in a reliable and reproducible manner represents an attractive alternative in centers without ready access to either advanced imaging modalities or stroke neurologists and/or neuroradiologists for imaging interpretation. Additionally, this approach offers other potential benefits that include (1) reduced contrast and radiation exposure and (2) faster times to treatment, which theoretically would lead to improved outcomes. Finally, this tool might become complementary rather than competing approaches. There were some limitations in this study. First was the retrospective nature and small sample size of this study. Second, our analysis lacked independent external validation. Hence, prospective studies are required with larger cohorts from external multicenter validation. Last, the semiautomatic methods to segment hemorrhage may be time-consuming. Future studies could explore the role of artificial intelligence in providing a rapid comprehensive segmentation (Zeng et al., 2018, 2021, 2022; Wu et al., 2022).

In summary, the NECT-based radiomics nomogram was developed and validated in this study, which provides a valuable tool for the individualized risk prediction of VMH in patients with ICH. As this information is not assessable by human eyes, the proposed approach can be used as a supportive tool to improve the radiologist’s diagnostic decision. Radiomics nomogram, as a non-invasive and quantitative method, may serve as a promising tool to complement the conventional procedures for the clinical decision-making process in future large-scale applications.

## Data Availability Statement

The original contributions presented in the study are included in the article/supplementary material, further inquiries can be directed to the corresponding author.

## Ethics Statement

The studies involving human participants were reviewed and approved by the Ethics Committee of Northern Jiangsu People’s Hospital. Written informed consent for participation was not required for this study in accordance with the national legislation and the institutional requirements.

## Author Contributions

Y-LY and JY guaranteed the manuscript. JW and XX contributed to conception, design, collection, and assembly of data. YY, JL, and JH involved in data analysis and interpretation. All authors contributed to the article and approved the submitted version.

## Conflict of Interest

The authors declare that the research was conducted in the absence of any commercial or financial relationships that could be construed as a potential conflict of interest.

## Publisher’s Note

All claims expressed in this article are solely those of the authors and do not necessarily represent those of their affiliated organizations, or those of the publisher, the editors and the reviewers. Any product that may be evaluated in this article, or claim that may be made by its manufacturer, is not guaranteed or endorsed by the publisher.

## Footnotes

1
http://www.slicer.org

## References

[B1] Artzi M. Bressler I. Ben B. D. (2019). Differentiation between glioblastoma, brain metastasis and subtypes using radiomics analysis. *J. Magn. Reson. Imaging* 50 519–528. 10.1002/jmri.26643 30635952

[B2] Barone D. G. Marcus H. J. Guilfoyle M. R. Higgins J. N. P. Antoun N. Santarius T. (2017). Clinical Experience and Results of Microsurgical Resection of Arterioveonous Malformation in the Presence of Space-Occupying Intracerebral Hematoma. *Neurosurgery* 81 75–86. 10.1093/neuros/nyx003 28328006

[B3] Choi Y. S. Rim T. H. Ahn S. S. Lee S. K. (2015). Discrimination of Tumorous Intracerebral Hemorrhage from Benign Causes Using CT Densitometry. *AJNR Am. J. Neuroradiol.* 36 886–892. 10.3174/ajnr.A4233 25634719 PMC7990598

[B4] Fukuda K. Majumdar M. Masoud H. Nguyen T. Honarmand A. Shaibani A. (2017). Multicenter assessment of morbidity associated with cerebral arteriovenous malformation hemorrhages. *J. Neurointerv. Surg.* 9 664–668. 10.1136/neurintsurg-2016-012485 27334979

[B5] Josephson C. B. White P. M. Krishan A. Al-Shahi Salman R. (2014). Computed tomography angiography or magnetic resonance angiography for detection of intracranial vascular malformations in patients with intracerebral haemorrhage. *Cochrane Database Syst. Rev.* 2014:CD009372. 10.1002/14651858.CD009372 PMC6544803 25177839

[B6] Kocak B. Yardimci A. H. Bektas C. T. Turkcanoglu M. H. Erdim C. Yucetas U. (2018). Textural differences between renal cell carcinoma subtypes: machine learning-based quantitative computed tomography texture analysis with independent external validation. *Eur. J. Radiol.* 107 149–157. 10.1016/j.ejrad.2018.08.014 30292260

[B7] Kolossvary M. Karady J. Kikuchi Y. Ivanov A. Schlett C. L. Lu M. T. (2019). Radiomics versus Visual and Histogram-based Assessment to Identify Atheromatous Lesions at Coronary CT Angiography: an ex Vivo Study. *Radiology* 293 89–96. 10.1148/radiol.2019190407 31385755 PMC6776230

[B8] Lambin P. Leijenaar R. Deist T. M. Peerlings J. de Jong E. E. C. van Timmeren J. (2017). Radiomics: the bridge between medical imaging and personalized medicine. *Nat. Rev. Clin. Oncol.* 14 749–762. 10.1038/nrclinonc.2017.141 28975929

[B9] Lorensen W. E. Johnson C. Kasik D. Whitton M. C. (2020). History of the Marching Cubes Algorithm. *IEEE Comput. Graph. Appl.* 40 8–15. 10.1109/MCG.2020.2971284 32149611

[B10] Ma C. Zhang Y. Niyazi T. Wei J. Guocai G. Liu J. (2019). Radiomics for predicting hematoma expansion in patients with hypertensive intraparenchymal hematomas. *Eur. J. Radiol.* 115 10–15. 10.1016/j.ejrad.2019.04.001 31084753

[B11] Meretoja A. Strbian D. Putaala J. Curtze S. Haapaniemi E. Mustanoja S. (2012). SMASH-U: a proposal for etiologic classification of intracerebral hemorrhage. *Stroke* 43 2592–2597. 10.1161/STROKEAHA.112.661603 22858729

[B12] Murthy S. B. Merkler A. E. Omran S. S. Gialdini G. Gusdon A. Hartley B. (2017). Outcomes after intracerebral hemorrhage from arteriovenous malformations. *Neurology* 88 1882–1888. 10.1212/WNL.0000000000003935 28424275 PMC5444313

[B13] Nawabi J. Kniep H. Kabiri R. Broocks G. Faizy T. D. Thaler C. (2020). Neoplastic and Non-neoplastic Acute Intracerebral Hemorrhage in CT Brain Scans: machine Learning-Based Prediction Using Radiomic Image Features. *Front. Neurol.* 11:285. 10.3389/fneur.2020.00285 32477233 PMC7232581

[B14] Shi Z. Zhu C. Degnan A. J. Tian X. Li J. Chen L. (2018). Identification of high-risk plaque features in intracranial atherosclerosis: initial experience using a radiomic approach. *Eur. Radiol.* 28 3912–3921. 10.1007/s00330-018-5395-1 29633002 PMC6081255

[B15] Shu Z. Fang S. Ding Z. Mao D. Cai R. Chen Y. (2019). MRI-based Radiomics nomogram to detect primary rectal cancer with synchronous liver metastases. *Sci. Rep.* 9:3374. 10.1038/s41598-019-39651-y 30833648 PMC6399278

[B16] Thabet A. M. Kottapally M. Hemphill J. C. III (2017). Management of intracerebral hemorrhage. *Handb. Clin. Neurol.* 140 177–194. 10.1016/B978-0-444-63600-3.00011-8 28187799

[B17] van Asch C. J. Velthuis B. K. Rinkel G. J. Algra A. de Kort G. A. Witkamp T. D. (2015). DIAGRAM Investigators. Diagnostic yield and accuracy of CT angiography, MR angiography, and digital subtraction angiography for detection of macrovascular causes of intracerebral haemorrhage: prospective, multicentre cohort study. *BMJ* 351:h5762. 10.1136/bmj.h5762 26553142 PMC4637845

[B18] Wagle W. A. Smith T. W. Weiner M. (1984). Intracerebral hemorrhage caused by cerebral amyloid angiopathy: radiographic-pathologic correlation. *AJNR Am. J. Neuroradiol.* 5 171–176. 6422718 PMC8332534

[B19] Wu P. S. Li H. Zeng N. Y. Li F. P. (2022). FMD-Yolo: an efficient face mask detection method for COVID-prevention and control in public. *Image Vis. Comput.* 117:104341. 10.1016/j.imavis.2021.104341 34848910 PMC8612756

[B20] Xie H. Ma S. Wang X. Zhang X. (2020). Noncontrast computer tomography-based radiomics model for predicting intracerebral hemorrhage expansion: preliminary findings and comparison with conventional radiological model. *Eur. Radiol.* 30 87–98. 10.1007/s00330-019-06378-3 31385050

[B21] Xu W. Ding Z. Shan Y. Chen W. Feng Z. Pang P. (2020). A Nomogram Model of Radiomics and Satellite Sign Number as Imaging Predictor for Intracranial Hematoma Expansion. *Front. Neurosci.* 14:491. 10.3389/fnins.2020.00491 32581674 PMC7287169

[B22] Zeng N. Y. Li H. Peng Y. H. (2021). A new deep belief network-based multi task learning for diagnosis of Alzheimer’s disease. *Neural. Comput. Appl.* 1–12. 10.1007/s00521-021-06149-6

[B23] Zeng N. Y. Qiu H. Wang Z. D. Liu W. B. Zhang H. Li Y. R. (2018). A new switching-delayed-PSO-based optimized SVM algorithm for diagnosis of Alzheimer’s disease. *Neurocomputing* 320 195–202. 10.1016/j.neucom.2018.09.001

[B24] Zeng N. Y. Wu P. S. Wang Z. D. Li H. Liu W. B. Liu X. H. (2022). A small sized object dection oriented multi-scale feature fusion approach with application to defect detection. *IEEE Trans. Instrum. Meas.* 71 1–14.

[B25] Zhang Y. Zhang B. Liang F. Liang S. Zhang Y. Yan P. (2019). Radiomics features on non-contrast-enhanced CT scan can precisely classify VMH-related hematomas from other spontaneous intraparenchymal hematoma types. *Eur. Radiol.* 29 2157–2165. 10.1007/s00330-018-5747-x 30306329

